# In vitro, cellular and in vivo studies of amyloid oligomers structure and toxicity: Challenges and advances

**DOI:** 10.1002/pro.70525

**Published:** 2026-03-12

**Authors:** Magdalena I. Ivanova, Carmelo La Rosa, Ayyalusamy Ramamoorthy

**Affiliations:** ^1^ Biophysics Program University of Michigan Ann Arbor Michigan USA; ^2^ Michigan Neuroscience Institute University of Michigan Ann Arbor Michigan USA; ^3^ Dipartimento di Scienze Chimiche Università degli Studi di Catania Catania Italy; ^4^ Department of Chemical and Biomedical Engineering, FAMU‐FSU College of Engineering Florida State University Tallahassee Florida USA; ^5^ National High Magnetic Field Laboratory Tallahassee Florida USA; ^6^ Institute Molecular Biophysics Florida State University Tallahassee Florida USA

**Keywords:** amyloid, biophysical methods, cellular models, disease, oligomers, polymorphism, protein aggregation, protein misfolding, structure

## Abstract

Oligomeric assemblies of amyloidogenic proteins, such as Aβ, tau, α‐synuclein, amylin, transthyretin, and TDP‐43, are increasingly recognized as key drivers of cellular dysfunction across a range of neurodegenerative and systemic disorders. However, their molecular properties remain poorly understood due to their low abundance, structural heterogeneity, and transient nature. This review outlines current methods for studying amyloid oligomers, including biophysical (NMR, cryo‐EM, HS‐AFM, mass spectrometry), computational (molecular dynamics simulations), and biological (cellular assays, organoids, and animal models) approaches. This review also covers emerging methods for detecting misfolded proteins within complex biological environments and live‐cell systems. Furthermore, we discuss recent advances that specifically address the challenges of studying oligomers, which are yielding crucial data on how these pathogenic species impair cellular homeostasis. Given the heterogeneity and transient nature of the oligomers, it is essential to utilize findings across diverse experimental platforms that yield complementary data and apply methods that also ensure reproducibility and mechanistic clarity with the goal of translating these findings into effective therapeutic strategies.

## 1 INTRODUCTION

Protein misfolding and aggregation are central to the pathogenesis of many diseases. Traditionally, it has been believed that amyloid fibrils, β‐sheet‐rich structures formed by the accumulation of misfolded protein subunits, are associated with cell death. However, our understanding of the toxic species formed by misfolded proteins has changed. A large body of evidence has suggested that transient oligomeric intermediates, which precede amyloid fibril formation, are the primary drivers of cellular dysfunction and death (Cardoso et al., 2010; Gurlo et al., 2010; Kao et al., 2015; Kayed et al., 2003; Larson & Lesné, 2012; Wells et al., 2021; Yang et al., 2017).

Studies of biologically relevant oligomers have been impeded by their inherent structural heterogeneity, low abundance, and kinetic instability (Cascella et al., 2022; Larson & Lesné, 2012; Rinauro et al., 2024; Stefani, 2012; Wei et al., 2025). Often, data acquisition using standard biophysical and biochemical methods is slow relative to the lifetime of the oligomers, resulting in data that averages over intermediates through which the oligomers evolved. Another drawback is that the high protein concentrations required for most experimental setups can artificially accelerate aggregation, potentially resulting in species that differ from those stable under physiologically relevant conditions (Choi & Dokholyan, 2021; Shea & Daggett, 2022; Simoneau et al., 2007). Studies of oligomeric species are further complicated by the fact that a single protein can form distinct oligomeric “strains”, each with unique structures and toxicities (Castillo‐Carranza et al., 2018; Glabe, 2008; Liu et al., 2021; Lo Cascio et al., 2025; Moore et al., 2023; Zampagni et al., 2011). Hence, experimental conditions highly influence the type and properties of the oligomeric species: in vitro preparations, which lack the complexity of cellular milieu, may fail to replicate disease‐relevant conformers, and ex vivo sample preparations may undergo purification‐induced conformational changes that result in altered conformations and toxicity (Broc et al., 2025; Lee et al., 2023). Furthermore, many biophysical methods, such as X‐ray diffraction, require the isolation of homogeneous oligomer mixtures, which can be technically challenging due to their instability. On the other hand, solution‐scattering approaches, such as small‐angle X‐ray scattering (SAXS), often coupled with size‐exclusion chromatography (SEC‐SAXS), can be used to study heterogeneous oligomer populations in solution directly and to extract molecular mass, radius of gyration, and low‐resolution shape. Thus, researchers must carefully optimize sample preparation and select analytical methods that account for potential changes in oligomeric species resulting from experimental handling.

In this review, we evaluate the strengths and limitations of many widely applied biophysical techniques, including NMR spectroscopy, X‐ray diffraction, cryo‐electron microscopy (cryo‐EM), high‐speed atomic force microscopy (HS‐AFM), mass spectrometry (MS), and molecular dynamics (MD) simulations, in determining the molecular structure, formation, and dynamics of oligomeric species. We also review the advantages and limitations of various cellular and animal models used to study oligomer‐induced pathology, including immortalized cell lines, patient‐derived induced pluripotent stem cell (iPSC) neurons, and transgenic mice. We discuss the need to select techniques that preserve and detect disease‐relevant oligomer states in biologically relevant systems. Each method has the unique capability of providing specific information about key properties of oligomers. Hence, by applying a combination of structural, biophysical, and cellular approaches, the researchers can more accurately define oligomer properties and their toxic impact on cells, a knowledge that is critical for the development of effective therapies for protein aggregation disorders.

## 2 METHODS FOR SIZE AND STRUCTURE DETERMINATION

Biochemical and biophysical techniques have provided key information on the structure, size, and stoichiometry, allowing us to connect structural information to toxicity function. However, the inherent instability, heterogeneity, and low abundance of the oligomers have delayed progress in our knowledge of oligomer structure and the acquired toxicity associated with protein misfolding. Many structural models of oligomers are derived by extrapolating from the known structures of their amyloid fibrils. However, this approach requires rigorous validation, especially given the variability of oligomeric species and the challenges of fast‐aggregating systems like IAPP. To overcome the inherent instability of oligomers, researchers often stabilize them for experimental studies; however, the biological relevance of these artificially stabilized species must be rigorously validated (Shea & Daggett, 2022).

### 2.1 The power and limitations of the biochemical and biophysical methods

The detection of misfolded oligomers often relies on their increased β‐sheet content, typically measured with dyes like ThT and Congo Red. However, these assays are non‐specific and require confirmation by spectroscopic methods like circular dichroism (CD) or Fourier‐transform infrared (FT‐IR). Critically, this approach can miss some pathogenic species, as certain toxic oligomers adopt non‐β‐sheet structures. TDP‐43 oligomers, for example, are recognized by conformation‐specific antibodies (OC, A11) but fail to produce a signal with β‐sheet‐sensitive dyes like ThT or Congo Red (Fang et al., 2014). This suggests that some toxic oligomers with atypical conformations can be undetected by conventional β‐sheet probes.

The toxicity of amyloid oligomers is often linked to increased surface hydrophobicity, which promotes non‐specific interactions with cellular membranes and accelerates fibril formation. Surface hydrophobicity is commonly probed with dyes such as 8‐anilinonaphthalene‐1‐sulfonic acid (ANS) and bis‐ANS, allowing researchers to detect oligomeric species and monitor conformational transitions (Fang et al., 2014; Limbocker et al., 2023; Mannini et al., 2014). Like other fluorescent dyes, ANS and bis‐ANS are sensitive to pH, lipids, and ionic strength. An additional limitation is their lack of specificity, as they can bind to hydrophobic regions on natively folded proteins.

The most common biophysical techniques used to study the secondary structures of oligomers and monitor conformational changes during fibril formation are CD, FT‐IR, and Raman spectroscopy (Arosio et al., 2015; Mannini et al., 2018). These methods have revealed that amyloid oligomers consist of β‐sheets, α‐helices, and disordered regions (Cerf et al., 2009; Fusco et al., 2017; Kirkitadze et al., 2001; Misra et al., 2016; Serra‐Batiste et al., 2016). CD and FT‐IR are simple and accessible methods for studying the secondary structure of oligomers. However, CD and FT‐IR studies use samples of oligomers prepared in vitro from recombinant proteins, which may not accurately reflect the structures of oligomers formed in crowded cellular environments with lipid membranes. Furthermore, both techniques yield ensemble‐averaged measurements, thereby obscuring heterogeneity within dynamic oligomer populations. Specifically, FT‐IR signals are sensitive to interference from water and common buffer salts (e.g., phosphate, Tris). While using D_2_O can resolve the H_2_O interference, it is also possible that the deuterium may alter the oligomer structure and aggregation. CD spectra, meanwhile, can be distorted by light scattering and require the use of buffers that do not contain compounds such as imidazole, DTT, or high‐salt concentrations, which absorb in the far‐UV region and interfere with the CD spectra. Still, CD and FT‐IR are widely applied and accessible methods for studying oligomers.

Raman spectroscopy is another spectroscopic technique that can probe oligomer secondary structure in situ and ex vivo. In live cells, Raman spectral imaging (RSI), based on spontaneous Raman scattering, can track conformational changes and aggregation over time. However, this technique gives relatively weak signals and has slower acquisition rates. Stimulated Raman scattering (SRS) is a coherent Raman modality that uses pump–Stokes beams and is a faster, more sensitive technique than RSI. SRS has been applied for studying label‐free studies of cells and tissues, including amyloid deposits in human brain tissue from Alzheimer's disease (AD) patients (Lochocki et al., 2021). The sensitivity of Raman methods can be increased by using silver nanoparticles, which amplify Raman signals, allowing the detection of concentrations as low as ~10 nM. This approach, known as surface‐enhanced Raman spectroscopy (SERS), has revealed, for example, the formation of heterogeneous IAPP/Aβ40 oligomers with a core‐shell structure (D'Urso et al., 2018).

Small‐angle X‐ray scattering (SAXS) is a solution‐based technique that measures X‐ray scattering at low angles, providing low‐resolution (~10–30 Å) structural information, including molecular mass, radius of gyration, and overall shape in solution (Kathuria et al., 2011; Kikhney & Svergun, 2015). This technique is useful for studying conformational changes during aggregation in real time (Lorenzen et al., 2014; Oliveira et al., 2009). However, SAXS is highly sensitive to sample polydispersity and heterogeneity. Hence, SAXS is often coupled with size‐exclusion chromatography (SEC) to separate oligomeric species before analysis. Data deconvolution and ensemble modeling are used to resolve the scattering contributions of individual species within a mixture (Blanchet et al., 2015; Mohammed et al., 2024). Although SAXS offers only low spatial resolution (~10–30 Å), it yields shape and distance constraints that help model how subunits assemble into oligomers, and it is often used to complement higher‐resolution methods such as NMR, X‐ray crystallography, and cryo‐EM (Lorenzen et al., 2014; Oliveira et al., 2009).

High‐speed atomic force microscopy (HS‐AFM) uniquely captures the transient dynamics of oligomeric species and membrane interactions, information inaccessible to ensemble‐averaged techniques like CD and FT‐IR. For example, using HS‐AFM, Ewald et al. directly observed Aβ_1–42_ pore formation and disruption of membranes containing ganglioside GM1 (Ewald et al., 2019; Feuillie et al., 2020). A key limitation, however, is that HS‐AFM is typically applied to simplified systems, which lack the complexity of a cellular environment. Hence, to gain a complete mechanistic understanding of oligomer toxicity, HS‐AFM findings need to be complemented by fluorescence‐based cellular assays and in vivo models.

Mass spectrometry (MS) provides information on oligomer size, stoichiometry, and structure. It can uniquely identify post‐translational modifications (PTMs) (Wang et al., 2018; Young et al., 2014). Native MS directly measures the mass of intact assemblies, from small oligomers like Aβ trimers to larger complexes (van den Heuvel & Heck, 2004). Aggregation pathways can be tracked by MS‐based kinetic studies in real time, such as the conversion of toxic Aβ oligomers into less toxic species (Lieblein et al., 2020). Tandem MS fragmentation can map specific PTMs. Despite challenges like gas‐phase dissociation and buffer interference (Hu & Zheng, 2020), MS is a valuable method for identifying distinct oligomeric species and elucidating the molecular interfaces that govern their assembly.

In summary, characterizing oligomers requires a multimodal approach, as no single technique provides complete information. Spectroscopic methods (ThT, CD, FT‐IR) report on bulk secondary structure but cannot resolve heterogeneous mixtures. SEC, dynamic light scattering (DLS), and AFM define size distributions but reveal little about atomic‐level conformation. Methods such as SAXS and native MS are ideal for studying oligomer dynamics, providing low‐resolution shape and stoichiometry data, and Raman techniques (SRS, RSI, SERS) allow in situ structural studies within complex biological samples.

### 2.2 NMR studies of amyloid oligomers

NMR spectroscopy has played a key role in understanding the structural and dynamic features of amyloid oligomers. While solution NMR spectroscopy is typically well‐suited for studying small‐sized oligomers and monomers, solid‐state NMR (ssNMR) techniques are applied to study large‐sized oligomers, protofibrils, and mature fibrils.

#### 2.2.1 Solution NMR spectroscopy

Solution‐state NMR spectroscopy fills a critical niche in amyloid research: it can characterize the transient and flexible nature of oligomers, properties inaccessible to many high‐resolution techniques (Dyson & Wright, 2021; Karamanos et al., 2015; Middleton, 2024; Toleikis et al., 2022; Waudby et al., 2013). It is particularly valuable for studying early oligomerization of intrinsically disordered proteins (IDPs) such as Aβ and α‐synuclein under near‐physiological conditions (Alderson & Kay, 2020; Cawood et al., 2022; Fu & Vendruscolo, 2015; Joshi & Vendruscolo, 2015; Matthes et al., 2016). For systems up to ~100 kDa, multidimensional NMR provides atomic‐level insights into secondary structure and backbone dynamics via relaxation measurements (T_1_, T_2_, heteronuclear NOE), and Carr–Purcell–Meiboom–Gill (CPMG) relaxation dispersion experiments can detect conformational exchange between monomers and oligomers on the microsecond‐to‐millisecond timescale, revealing the initial steps of aggregation (Korzhnev et al., 2004; *Nat Protoc*, 2012; Torricella et al., 2024; Tugarinov et al., 2022).

Common NMR experiments for mapping aggregation‐prone regions include chemical shift perturbation (CSP) analysis, which studies interaction interfaces that form the oligomers by monitoring chemical shift changes during the titration of monomeric protein with oligomeric species or external modulators. β‐Sheet formation or buried hydrophobic cores in oligomers can be studied by hydrogen/deuterium exchange (HDX) experiments, which identify regions protected from solvent exchange (Maiti et al., 2024; Nguyen et al., 2022). Additionally, paramagnetic relaxation enhancement (PRE) with site‐directed spin labeling of proteins informs on the long‐range distance restraints that reveal transient intra‐ or intermolecular contacts in low‐populated oligomeric states. Data on the hydrodynamic radii and real‐time oligomerization can be obtained from diffusion‐ordered spectroscopy (DOSY) (Kandiyal et al., 2019; Pagano et al., 2024; Xu et al., 2025). Rheo‐NMR monitors structural and dynamic changes in proteins while applying mechanical stress (shear or flow) directly to protein solutions, and it has been used to investigate how proteins misfold and aggregate (Iwakawa et al., 2017; Iwakawa et al., 2021; Morimoto et al., 2022). Another method, fluorine‐19 NMR, relies on fluorine labels, which are not present in the natural proteins and therefore provide site‐specific, background‐free signals that are highly sensitive to their local environment. Monitoring changes in ^19^F chemical shifts and line shapes allows researchers to detect conformational changes and transient oligomer formation during the early stages of aggregation (Duan et al., 2022; Jack et al., 2006; Sahoo, Cox, & Ramamoorthy, 2020; Suzuki et al., 2012; Suzuki et al., 2013; Takaoka et al., 2013; Toleikis et al., 2022; Tooyama et al., 2016; Wadhwani et al., 2012; Yanagisawa et al., 2011; Yanagisawa et al., 2014). Dark‐state exchange saturation transfer (DEST) and relaxation‐based methods have quantified exchange with low‐populated or “dark” states and protofibril contacts at atomic resolution and interactions with chaperones (Fawzi et al., 2010; Fawzi et al., 2014; *Nat Protoc*, 2012). These combined approaches are useful to dissect structural polymorphism, assembly pathways, and intermediate states in aggregation.

NMR methods have been essential in understanding the Aβ aggregation, giving insights into the transitions of monomers into β‐sheet–rich oligomers (Niu et al., 2024; Pagano et al., 2024; Wälti et al., 2015; Wälti, Orts, & Riek, 2017), the formation of micelle‐like states (Wälti et al., 2015), and the effects of salt, metal ions, and pH on monomer‐aggregate equilibria (Narayanan & Reif, 2005; Rezaei‐Ghaleh et al., 2019). Relaxation‐based and DEST NMR techniques have resolved transient exchange processes between soluble and protofibrillar states (Brender et al., 2019; Fawzi et al., 2010; Fawzi et al., 2014; *Nat Protoc*, 2012). Methyl‐resolved methods have mapped aggregation‐prone cores and side‐chain dynamics (Prade et al., 2015; Wälti, Orts, & Riek, 2017). Small molecules, including epigallocatechin gallate (EGCG), nonsteroidal anti‐inflammatory drugs (NSAIDs), and sulindac sulfide, have also been studied by solution NMR to show their ability to modulate Aβ aggregation by altering aggregation pathways or interacting with oligomer‐forming surfaces (Braymer et al., 2011; Cox et al., 2020; del Amo et al., 2012; Prade et al., 2015). Solution NMR has shown how residue‐specific modifications (e.g., phosphorylation, Met35 oxidation) alter aggregation (Friedemann et al., 2015), and how cross‐interactions with IAPP‐blocker peptides (Rezaei‐Ghaleh et al., 2011) or molecular chaperones such as Hsp104 (Ghosh, Tugarinov, & Clore, 2024; Tugarinov et al., 2025) and GroEL (Wälti et al., 2018; Wälti et al., 2021; Wälti, Schmidt, et al., 2017) regulate Aβ fibril formation.

In addition to Aβ, solution NMR has been successfully applied to study the aggregation of other proteins. For α‐synuclein, solution NMR revealed membrane‐bound conformations of disease‐relevant mutations (Buratti et al., 2022; Buratti et al., 2023; Cho et al., 2011), monomer–fibril binding, secondary nucleation mechanisms, and lipid co‐assembly dynamics (Ghosh, Torres, et al., 2024; Kumari et al., 2021). In combination with ssNMR, HDE studies further provided the atomic‐level structure of α‐synuclein fibrils (Vilar et al., 2008). Tau protein condensation and aggregation have been shown to depend on post‐translational modifications and co‐chaperone networks involving FKBP51 and p23 (Chakraborty et al., 2023; Chakraborty & Zweckstetter, 2025). Similarly, solution NMR has been used to study transient oligomeric species of huntingtin exon‐1 aggregation and their interactions with chaperones (Kotler et al., 2019; Wälti et al., 2021).

Functional amyloids, such as β‐endorphin fibrils and bacterial biofilm components (FapC, FapA), have been analyzed with solution NMR, revealing highly dynamic assembly–disassembly behavior and secondary structure propensities (Fawzi et al., 2011; Nespovitaya et al., 2016). Solution NMR has also been used to study the effects of small molecules, lipids, insulin, zinc, and pH on IAPP aggregation (Bhowmick et al., 2022; Garcia‐Vinuales et al., 2022; Khemtemourian et al., 2021; McCalpin et al., 2024; Nanga et al., 2011; Pithadia et al., 2016; Rodriguez Camargo et al., 2017; Taylor et al., 2025). Functional SEVI amyloids (derived from PAP248‐286), which enhance viral infection, have also been characterized using solution NMR (Brender et al., 2011; Nanga et al., 2009).

#### 2.2.2 Solid‐state NMR spectroscopy

Solid‐state NMR spectroscopy is widely used to investigate the structures, dynamics, and assembly of amyloid oligomers (Dasari & Lim, 2022; Hiroaki, 2023; Jeon et al., 2023; König et al., 2021; Kotler et al., 2015; Niu et al., 2024; Scheidt et al., 2012; Scheidt et al., 2020; Takamuku et al., 2022; Yeh et al., 2023). Using magic‐angle spinning (MAS) to average anisotropic interactions, together with isotopic labeling, ssNMR allows assignment of ^13^C and ^15^N chemical shifts that provide information on secondary structure. Intermolecular distances can be probed through recoupling MAS experiments, such as DARR (dipolar‐assisted rotational resonance), PITHIRDS‐CT (Tycko, 2007), and REDOR (rotational‐echo double resonance), to provide long‐range constraints defining inter‐residue and intermolecular contacts (Tycko, 2007). These data are then used to model aggregate structures and distinguish between different amyloid polymorphs.

ssNMR provides site‐specific and residue‐level resolution for heterogeneous aggregates (Kengwerere et al., 2025; Meredith, 2006; Tay et al., 2013; Townsend et al., 2018; Walsh et al., 2014; Xiao et al., 2020). Studies of Aβ oligomers have revealed out‐of‐register parallel and antiparallel β‐sheets (Chang et al., 2024; Hora et al., 2017; Lee et al., 2018; Qiang et al., 2020), conformational changes due to posttranslational modifications, such as pyroglutamate formation (Gao et al., 2020; Scheidt et al., 2020), and by metal ions (Kechko et al., 2023; Lee et al., 2025; Mannini et al., 2018; Xie et al., 2020). ssNMR analyses of Aβ (Adlakha, 2023; Al Adem & Lee, 2023; Alderson & Kay, 2020; Amin & Harris, 2021; Andrews et al., 2025; Apostol et al., 2013; Arbel‐Ornath et al., 2017; Arosio et al., 2015; Babapour Mofrad et al., 2021; Barredo & Balanay, 2023; Bhowmick et al., 2022; Binolfi et al., 2012; Blanchet et al., 2015; Borbat & Freed, 2013; Braymer et al., 2011; Brender et al., 2011; Brender et al., 2019; Broc et al., 2025; Bukhari & Müller, 2019; Buratti et al., 2022; Buratti et al., 2023; Cabral et al., 2022; Cardoso et al., 2010; Cascella et al., 2021; Cascella et al., 2022; Castillo‐Carranza et al., 2018; Cawood et al., 2022; Cehlar et al., 2024; Cerf et al., 2009; Cerneckis et al., 2024; Chakraborty et al., 2023; Chakraborty & Zweckstetter, 2025; Chang et al., 2022; Chang et al., 2024; Chen et al., 2015; Cheng, 2015; Cho et al., 2011; Choi & Dokholyan, 2021; Chou et al., 2018; Coles et al., 2025; Costello et al., 2019; Cox et al., 2020) have provided information on structural transitions from soluble spherical oligomers to fibrils (Walsh et al., 2009), cross‐seeding between Aβ40 and Aβ42 (Chang et al., 2022), and conformational changes driven by lipid and detergent micelles (Chang et al., 2024; Kumar et al., 2016; Lendel et al., 2014). ssNMR findings added to our understanding of on‐pathway and off‐pathway oligomers (Cruceta et al., 2023) and the mechanisms of membrane disruption (Kenyaga et al., 2022; Korshavn et al., 2017). ssNMR has also provided structural detail on tau, α‐synuclein, hIAPP, transthyretin, and prion protein oligomers (Cehlar et al., 2024; Dasari & Lim, 2022; Hiroaki, 2023; Saha et al., 2025; Takamuku et al., 2022; Zhao et al., 2011a; Zhao et al., 2011b). For example, dynamic nuclear polarization‐enhanced ssNMR has followed the conformational evolution of α‐synuclein from liquid–liquid phase separation to amyloid fibrils (Takamuku et al., 2022), while MAS‐based studies of hIAPP have revealed polymorphic oligomers (McCalpin et al., 2023). Prion protein‐derived peptides have likewise been shown to form distinct β‐rich oligomers and interact with membranes (Daskalov et al., 2021; Helmus et al., 2010; Li et al., 2022; Loquet et al., 2018; Qi et al., 2022; Theint et al., 2017). In addition, ssNMR has demonstrated that small molecules such as EGCG remodel toxic fibrils into soluble oligomers (Middleton, 2024). To obtain more complete data on oligomer structures, ssNMR studies are often complemented by CD spectroscopy, light scattering, SAXS, and analyses of side‐chain dynamics (Dasari & Lim, 2022; van der Wel, 2018).

Despite its strengths, ssNMR can be technically demanding, requiring large amounts of isotopically labeled samples. Highly heterogeneous and polymorphic samples can also limit the sensitivity and spectral resolution. However, some of these challenges can be overcome with a combination of fast‐MAS, non‐uniform sampling, proton‐detection, and DNP (dynamic nuclear polarization) methods (Elathram et al., 2022; Le Marchand et al., 2022). Many reviews and methodological papers emphasize the power of NMR in resolving amyloid assembly pathways, molecular kinetics, and inhibitor actions, providing tools to dissect transient conformers inaccessible to crystallography or cryo‐EM (Karamanos et al., 2015; Middleton, 2024).

#### 2.2.3 In‐cell NMR for studying the dynamics of protein misfolding and aggregation

In‐cell NMR spectroscopy has been developed to directly observe proteins in living cells and to gain insights into dynamic post‐translational modifications and degradation pathways (Freedberg & Selenko, 2014; Ikeya et al., 2010; Luchinat et al., 2021; Sakakibara et al., 2009; Separovic et al., 2022; Serber et al., 2006; Theillet & Luchinat, 2022; Thongwichian & Selenko, 2012). A key application has been the study of intrinsically disordered proteins like α‐synuclein. Time‐resolved in‐cell NMR further showed that α‐synuclein undergoes regulated proteolytic processing both in vitro and in vivo (Binolfi et al., 2012; Waudby et al., 2013). Time‐resolved in‐cell NMR has further revealed that α‐synuclein undergoes regulated proteolytic processing and phosphorylation‐dependent degradation within cells (Limatola et al., 2018; Zheng et al., 2019).

In‐cell NMR has also proven valuable for studying the aggregation of SOD1, a protein that misfolds in ALS. Strategic protein engineering of ALS‐linked SOD1 mutants improved spectral resolution sufficiently to enable the detection of disease‐relevant misfolding (Danielsson et al., 2013). Subsequent work showed that the copper chaperone CCS can rescue the folding of these mutants in human cells (Luchinat et al., 2017). Advances in delivery, labeling, and methods like DNP‐enhanced NMR now allow tracking of protein maturation and folding at near‐endogenous concentrations in human cells (Costello et al., 2019; Luchinat & Banci, 2018), the power of in‐cell NMR for real‐time monitoring of functional rescue.

### 2.3 X‐ray crystallography of amyloid‐like oligomers

X‐ray diffraction has provided high‐resolution insights into amyloid oligomers, revealing structural motifs distinct from mature fibrils that are associated with toxicity (Apostol et al., 2013; Laganowsky et al., 2012; Spencer et al., 2014; Stroud et al., 2012). Examples include the “cylindrin” oligomer, a compact, six‐stranded antiparallel β‐barrel with a central hydrophobic channel formed by an αB‐crystallin segment (Laganowsky et al., 2012), and oligomers from Aβ‐derived peptides that assemble into β‐hairpin‐based trimers with out‐of‐register β‐sheets (Spencer et al., 2014). While X‐ray diffraction provides atomic‐resolution data, crystallization requires stable, homogeneous samples. Hence, X‐ray diffraction structures often reflect only the most ordered species, representing a limited subset of the dynamic oligomer populations present in solution and the living cells.

### 2.4 Cryo‐electron microscopy

Cryo‐electron microscopy (cryo‐EM) has much potential for gaining high‐resolution structural insights into oligomeric species (Chen et al., 2015; Dasari et al., 2025; Lövestam et al., 2024; Wu et al., 2021). However, because oligomers are often small (<100 kDa), heterogeneous, and transient, single‐particle cryo‐EM studies are limited by low signal‐to‐noise and difficulties in particle alignment due to structural variability (Lander & Glaeser, 2021). To overcome limitations due to low oligomer concentrations and heterogeneous samples, an antibody‐based method has been developed that directly immobilizes oligomers onto antibody‐functionalized grids before vitrification. This method also stabilizes fragile subpopulations, reducing background and helping capture rare species. To enhance resolution, improved 3D classification algorithms have also been developed to separate species with different conformations (Cabral et al., 2022; Zhang et al., 2008; Zhao et al., 2024). Additional approaches, such as cryo‐electron tomography (cryo‐ET) and time‐resolved cryo‐EM, are commonly used to study oligomers in tissue or cellular samples, eliminating the need to extract and isolate the oligomers.

### 2.5 Electron paramagnetic resonance spectroscopy

Electron paramagnetic resonance (EPR) spectroscopy has long been applied to characterize amyloid fibrils formed in solution and in membrane‐mimetic environments (Der‐Sarkissian et al., 2003; Karyagina et al., 2011; Tao et al., 2019; Török et al., 2002; Varkey & Langen, 2017). More recently, due to its high sensitivity and ability to provide site‐specific dynamics and distance restraints, EPR has been applied for studies of short‐lived and heterogeneous amyloid oligomers (Gu & Guo, 2021; Jang et al., 2023; Sepkhanova et al., 2009; Zurlo et al., 2019; Zurlo et al., 2022). Based on EPR measurements, Jang et al. identified three structured segments spanning residues 9–11, 15–22, and 30–40 that adopt fibril‐like conformations in Aβ42 oligomers (Jang et al., 2023). Intermolecular spin–spin interactions were consistent with a parallel, in‐register β‐sheet organization, supporting the existence of a fibril‐like subclass of Aβ42 oligomers (Jang et al., 2023).

Among the caveats of EPR is the need for protein labeling with paramagnetic spin labels (Margittai & Langen, 2006), which can alter the aggregation process. Hence, comparative studies of aggregated unlabeled and labeled proteins are necessary. Furthermore, many EPR experiments, particularly pulsed EPR distance measurements (DEER/PELDOR), require efficient labeling and concentrated samples of tens of micromolar concentrations (Borbat & Freed, 2013). EPR gives information about site‐specific dynamics, local environments, and nanometer‐scale distance distributions in oligomeric samples, and its strength lies in its use together with techniques that characterize oligomer assemblies, such as SEC‐SAXS, native MS, or AFM.

### 2.6 In‐cell studies with integrated biophysical and imaging methods

Promising techniques which have the potential to give both structural and functional information on oligomers within cells are the super‐resolution fluorescence imaging techniques, such as stimulated emission depletion microscopy (STET), structured illumination microscopy (SIM), and single‐molecule localization microscopy (SMLM) microscopy (Orte et al., 2008; Pickett et al., 2016; Sun et al., 2024). Applying these techniques made it possible to observe transient species and infer their toxic function in cellular models, where oligomers are detected with fluorescent dyes (e.g., ThT and Nile Blue) or conformation‐specific antibodies in cells and tissues (Esparza et al., 2013; Sun et al., 2024; Tomic et al., 2009). However, the fluorescent probes can perturb the oligomer structure or dynamics, and imaging artifacts can complicate data interpretation. Additionally, the higher resolution (~20–60 nm) of the super‐resolution methods is achieved at the cost of significantly longer acquisition times and reduced sensitivity.

Förster resonance energy transfer (FRET) is another live‐cell fluorescence method that can be used for monitoring oligomer formation and population dynamics in real time (Kjaergaard et al., 2018). In FRET, the protein of interest is tagged with donor and acceptor fluorophores. When donor–acceptor pairs approach within ~1–10 nm, non‐radiative energy transfer occurs and the FRET readout reports molecular proximity. A limitation, however, is the high sensitivity to both the distance (∝ 1/𝑟^6^) and the relative dipole orientation of the fluorophores, which can introduce ambiguity into distance measurements. Hence, controls for spectral bleed‐through, direct acceptor excitation, donor quenching, and donor–acceptor stoichiometry need to be carefully designed. Consequently, FRET data require cautious interpretation and are typically validated with super‐resolution imaging or biochemical assays.

Photo‐induced cross‐linking of unmodified proteins (PICUP) is a relatively simple assay for studying oligomers in cellular contexts. Its key advantage is the ability to rapidly stabilize transient oligomeric species by cross‐linking without requiring fluorescent labels or protein modifications. When combined with SDS‐PAGE, mass spectrometry, or Western blotting, PICUP can be used for the determination of oligomer size and identity of the misfolded protein in samples derived from cell cultures or animal tissues and is useful for studying early aggregation intermediates (Amin & Harris, 2021; Frangolho et al., 2020; Lassen et al., 2018; Roberts et al., 2015). However, non‐specific cross‐linking between the protein of interest and other cellular proteins can create artifacts and complicate the interpretation of oligomer size.

A combination of cryo‐EM with fluorescence microscopy has emerged as a powerful approach for studying oligomers. For example, cryogenic correlative light and electron microscopy (cryo‐CLEM) and cryo‐ET were used to visualize the mislocalization and aggregation of TDP‐43 within subcellular compartments of neurons derived from patients with ALS/FTD (Erwin et al., 2024). Similarly, Guo et al., using cryo‐ET and aided by light microscopy, revealed that GA dipeptide repeat aggregates form densely packed ribbons that stall 26S proteasomes and disrupt proteostasis in neuronal cell culture models of ALS/FTD (Guo et al., 2018). These examples show that a combination of cryo‐EM with fluorescent methods and cellular or in vivo disease models can capture oligomer structures in situ and inform on their toxic function.

### 2.7 MD simulations reveal transient structure and toxic interactions of amyloid oligomers

Molecular dynamics (MD) simulation is an in silico technique that can provide insights into the heterogeneous conformational changes of short‐lived oligomeric species, details often inaccessible to experimental methods. Applications of MD span early stages of oligomer assembly (Redler et al., 2014; Tarus et al., 2008), oligomer interactions with lipid membranes, metal ions, and molecular chaperones (Mutter et al., 2020; Qian et al., 2016; Zhang et al., 2007), the effects of post‐translational modifications on oligomer stability (Barredo & Balanay, 2023), and pore formation in membranes and ligand binding (Matthes & de Groot, 2023; Tsigelny et al., 2008). However, the predictive power is primarily constrained by the accuracy of its force fields and the limited timescales of the simulation. Furthermore, the findings of this in silico method require experimental validation.

## 3 DETECTING MISFOLDED PROTEINS IN COMPLEX BIOLOGICAL MILIEUS

Analysis of postmortem human and in vivo models tissues provides information on the later stages of pathogenic aggregation and the oligomeric species in these tissues are commonly detected with conformation‐specific immunohistochemistry (e.g., A11, OC), immunogold EM, and dot blot assays (Al Adem & Lee, 2023; Fá et al., 2016; Funke, 2011; Lo Cascio et al., 2025).

Enzyme‐linked immunosorbent assays (ELISA) are standard for protein quantification in complex biofluids, but this method suffers from low sensitivity for oligomers due to masked epitopes (Babapour Mofrad et al., 2021; Janssen et al., 2015; Peden et al., 2012). In addition, oligomers and fibrils can have inefficient or variable adsorption to ELISA plate surfaces, and complex sample matrices (e.g., brain homogenates or crude plasma) can further reduce immobilization and increase nonspecific interactions, limiting detection sensitivity. To overcome these limitations, time‐resolved immunofluorometric assays (TRIFMAs) have been developed to optimize the spacing between the antibodies (Holme et al., 2022). To minimize monomer interference, multimer‐detection systems pair capture antibodies that bind all protein species with detection antibodies specific for oligomeric epitopes (Jamerlan et al., 2025).

Flow cytometry‐based methods provide another alternative for analysis of complex biological samples and can distinguish oligomeric particles in plasma by size and antibody labeling (Dayarathna et al., 2024; Santos et al., 2008). Still, a limitation of the antibody‐based technique is cross‐reactivity between the antibody and unrelated proteins (Kayed et al., 2003; Kumar et al., 2020). Additionally, there is a trade‐off between specificity and sensitivity: while antibodies with high specificity reduce cross‐reactivity, they often produce low signals due to weaker binding affinity (Kumar et al., 2020).

Proximity ligation assays (PLAs) have been applied for direct, in situ detection of oligomers in tissue specimens. In this method, antibodies are conjugated to complementary DNA strands that, when brought within ~40 nm, are ligated together. Subsequent DNA amplification generates a strong fluorescent or chromogenic signal (Söderberg et al., 2008). For example, utilization of PLA has successfully identified endogenous oligomeric α‐synuclein in postmortem brain tissue and revealed early pathology not detected by conventional immunohistochemistry (Roberts et al., 2015). The PLA signal can be attenuated in late‐stage aggregates by epitope masking, and the PLA signal heavily depends on epitope proximity, density, accessibility, and amplification, not just oligomerization. Hence, PLA is commonly complemented by ELISA, immunogold EM, and mass spectrometry to confirm oligomer identity.

Elevated levels of amyloid proteins and their oligomers in CSF and plasma are well‐established biomarkers for AD and PD disease (Delaby et al., 2023; Wang et al., 2023). Seed‐amplification assays, such as RT‐QuIC and PMCA, can detect femtomolar to attomolar concentrations of these misfolded oligomers in CSF with high sensitivity by using the ability of prion oligomers to template the aggregation of recombinant substrates (Paciotti et al., 2018; Peden et al., 2012; Singh & DeMarco, 2020; Vascellari et al., 2022). These assays can distinguish α‐synuclein, tau, and prion seeds in CSF (Dong & Satoh, 2021; Kuang et al., 2024; Mok et al., 2021; Scialò et al., 2020; Vascellari et al., 2022). Despite the general challenge of strain discrimination, this technology has been successfully applied to differentiate α‐synuclein strains associated with distinct synucleinopathies, suggesting its potential application for pathological subtyping (Shahnawaz et al., 2020). Although these assays have been successfully used for CSF, their robust application to peripheral biofluids like plasma is still an area of active development (Vascellari et al., 2022).

## 4 CELLULAR MODELS

### 4.1 Toxicity studies in immortalized cell lines

Research into oligomer‐induced toxicity uses a range of cellular models, from immortalized lines (e.g., SH‐SY5Y) for high‐throughput screening to more complex patient‐derived induced pluripotent stem cells (iPSCs). MTT (3‐(4,5‐dimethylthiazol‐2‐yl)‐2,5‐diphenyl‐tetrazolium bromide, metabolic activity) and LDH (lactate dehydrogenase, membrane integrity) are among the standard assays for initial screening of metabolic viability and cytotoxicity, respectively. The results from these assays, however, require careful interpretation (Cox et al., 2020; Fändrich, 2012; Kruger et al., 2020; Sahoo, Bekier, et al., 2020; Shearman et al., 1994; Sun et al., 2014). For example, Aβ oligomers can artificially accelerate MTT formazan exocytosis, leading to a false‐positive increase in the readout signal, potentially masking underlying toxicity (Liu & Schubert, 1997). An absence of signal in MTT assays does not always indicate a lack of toxicity, as some immunoglobulin light chain (LC) oligomers induce caspase‐mediated apoptosis without producing a signal in MTT assays (Sengupta et al., 2016). Therefore, using a single test to detect toxicity is insufficient, and the initial toxicity screening must employ a panel of assays that probe distinct cellular pathways.

### 4.2 Exogenous oligomer application

Although many amyloid proteins, such as α‐synuclein and tau, are normally intracellular, the external application of their preformed oligomers has been instrumental in studying their toxicity. The external application of oligomers to immortalized cell lines (e.g., HEK293, SH‐SY5Y, Neuro‐2a) has elucidated multiple mechanisms of toxicity, including lysosomal impairment, membrane lipid remodeling, oxidative stress, mitochondrial dysfunction, and proteostasis failure (Cascella et al., 2021; Coles et al., 2025; Delenclos et al., 2019; Klucken et al., 2012; Outeiro et al., 2008). Many of the findings in immortalized cells have also been reproduced in more complex neuronal and in vivo models (Arbel‐Ornath et al., 2017; Hoffmann et al., 2019; Kuchibhotla et al., 2008; Lee et al., 2022; Lerdkrai et al., 2018).

FRET‐based biosensor cell lines are among the widely used assays to study oligomer uptake and the intracellular spread of misfolded species (Holmes & Diamond, 2017; Maina et al., 2022). The biosensor cells, typically derived from human HEK‐293 cells, express tau or α‐synuclein tagged with fluorescent proteins (CFP and YFP). In these cells, the internalization of externally applied oligomers triggers the aggregation of the labeled proteins, which brings the fluorophore labels close enough to generate a FRET signal quantifiable by microscopy or flow cytometry. Because the label can interfere with oligomer formation, the focus of biosensor cell development has been on using smaller fluorophores as labels and performing rigorous comparative studies to confirm that tagging does not artificially alter oligomer properties (Evans et al., 2018; Haney et al., 2016; Kaniyappan et al., 2020).

The pathological relevance of oligomer toxicity is intrinsically tied to the native subcellular localization of the precursor proteins. Thus, the use of proteins whose functions depend on location is inherently limited for external applications and may result in toxic effects that differ from those of aggregates formed in vivo. For instance, the mislocalization of proteins like TDP‐43 and FUS is a known trigger for their aggregation and toxicity, and disruptions to the nuclear‐cytoplasmic transport machinery can either promote or suppress the formation of toxic oligomers (Chou et al., 2018; Kinger et al., 2023; Lin et al., 2021; Shang et al., 2017).

While exogenous application of oligomers (e.g., α‐synuclein, tau) offers precise dosing and timing, the approach faces limitations. Recombinant preparations may not recapitulate the structural heterogeneity present in human disease, and tissue‐derived oligomers can be altered by harsh extraction protocols (Varshavskaya et al., 2022). Moreover, because oligomers are transient and labile, experimental conditions, such as buffer composition, temperature, and incubation time, can alter their structure and kinetics (Dear et al., 2020), complicating data interpretation, particularly in toxicity MTT and LDH assays (Choi & Dokholyan, 2021; Simoneau et al., 2007). Therefore, these findings need further validation.

### 4.3 Overexpression systems

Overexpression systems in immortalized and neuronal cells have been popular for studying protein misfolding due to their convenience and reproducibility. However, their non‐physiological protein levels can generate artifacts. An alternative to overexpression is labeling endogenous proteins with fluorescent tags by CRISPR/Cas9 and monitoring their oligomer formation with single‐molecule fluorescence imaging under native conditions (Bukhari & Müller, 2019; Haupt et al., 2018; Sun et al., 2024). A key consideration is that even small tags can influence the aggregation kinetics and potentially skew interpretations (Haney et al., 2016; Shimogawa & Petersson, 2021).

### 4.4 Primary neuronal cultures and patient‐derived iPSC neurons

While differentiating cells like SH‐SY5Y improves their relevance for studying the aggregation of some proteins, such as α‐synuclein (Xin et al., 2015), these models remain limited by inherent instability. Hence, primary rodent hippocampal neurons have become a preferred cellular model with greater physiological relevance. For example, studies using these cultures have shown that Aβ oligomers acutely disrupt synaptic function, cause calcium dysregulation, oxidative stress, synaptic loss, and cell apoptosis (De Felice et al., 2007; Lee et al., 2022; Li et al., 2011).

The use of patient‐derived iPSC neurons has increased because they provide a genetically matched system for investigating disease mechanisms. For example, key insights about α‐synuclein toxicity, such as defects in chaperone‐mediated autophagy and endoplasmic reticulum stress, have been gained using iPSC neurons (Drouin‐Ouellet, 2022 #159; Heman‐Ackah, 2017 #201; Bellucci, 2011 #203). The primary constraint with this cellular model is inter‐donor variability, which can confound the interpretation of disease‐relevant phenotypes (Cerneckis et al., 2024; Kyttälä et al., 2016). Hence, lines from multiple patients should be used when possible, along with the incorporation of isogenic controls or CRISPR‐corrected lines to separate genotype‐specific effects from background variation (Germain & Testa, 2017). Additionally, the lack of a native tissue context, including cell–cell interactions and extracellular matrix, can amplify oligomer toxicity and alter its behavior (Fändrich, 2012), necessitating further validation in organoids or animal models.

### 4.5 3D organoid cultures for tissue‐relevant context

3D organoid cultures recreate many aspects of brain structure, including cell‐type diversity, layered architecture, and networked interactions. A notable advance is the development of a vascularized neuro‐immune organoid that, when exposed to brain extracts from sporadic AD patients, accumulated Aβ‐ and tau‐like inclusions, displayed synaptic loss, and neuroinflammation within 4 weeks, thereby closely mirroring human tissue pathology (Ji et al., 2025). However, diffusion limitations in thick tissues lead to nutrient/oxygen deprivation and central necrosis during prolonged incubations. Hence, maintaining organoids beyond ~2–3 months to model aging is difficult, constraining studies of late‐onset disease (Kim & Chang, 2023). Achieving the right mix of neurons and glia to promote vascularization and reliable growth remains challenging, and each new batch needs to be verified, making the growth of these cultures time and resource‐demanding (Adlakha, 2023). Moreover, reproduction of myelination and a functional blood–brain barrier, as well as a mature extracellular matrix is still in the development stage. In summary, successful maintenance of organoid culture requires consistent cell differentiation, minimizing central necrosis, and incorporating missing cell types. Their strength, however, lies in their ability to model disease pathology and capture in vivo‐like complexity.

## 5 CONCLUSION AND FUTURE PERSPECTIVES

Here, we review the techniques commonly applied to study amyloid oligomers and their pathogenic roles in cells. Specifically, experimental strategies discussed in this review (summarized in Table 1) are aimed at addressing three critical oligomer properties: structural polymorphism, transiency, and biological relevance, and to address questions such as: (i) What species exist and in what distribution? (size/stoichiometry and abundance); (ii) What are the structural features of the oligomers?; (iii) How do oligomers form and interconvert? (kinetics of formation); (iv) What is their localization and distribution in cells?; and (v) Which species are most closely linked to toxic phenotypes?

**TABLE 1:** Methods summery for oligomer studies.

Method	Structural polymorphism^a^	Transiency^a^	Biological relevance^a^	Resolution^b^	Comments
Cryo‐EM	High	Low	Medium	~3–5 Å (Cheng, 2015)	Challenging for proteins with disordered regions such as oligomers. Larger resolutions require larger number of homogeneous oligomers. Sizes larger than ~50 kDa
Cryo‐ET	Medium	Medium	High	Wide range (~3–50 nm or larger) (Hutchings & Zanetti, 2018; Mahamid et al., 2016; Sun et al., 2022; Tegunov et al., 2021)	Commonly coupled with cryo‐CLEM to visualize cellular content. Limited by sample thickness and heterogeneity
ssNMR	High	Medium	Medium	~3–5 Å for fibrils (*Annu Rev Phys Chem*, 2011) Residue‐level for oligomers (Kotler et al., 2015)	Requires isotope labeling and homogeneous sample; no size limitation; enable dynamics measurements
Solution NMR	Medium to high	High	Low to medium	Residue‐level resolution (Sahoo, Cox, & Ramamoorthy, 2020)	Excellent for transient and early oligomers; limited to <100 kDa species; provides information on local contacts of the oligomers; enable dynamics measurements
EPR	Medium to high	High	Low to medium	distance restraints (~1.5–8 nm (DEER/PELDOR))	Excellent for transient and early oligomers; requires sire‐specific spin‐label (Borbat & Freed, 2013)
HS‐AFM	Medium	High	Low to medium	~2–3 nm lateral (Umeda et al., 2023)	Captures dynamic morphology; works in fluid
Super‐resolution microscopy	Low to medium	High	High	~20–60 nm	Requires labeling with fluorescent tags; artifacts can skew data interpretation
FRET	Low	High	High	~1–10 nm (distance sensitivity)	Requires labeling with fluorescent tags; ensemble averaging may mask heterogeneity
Single‐molecule fluorescence	Medium	High	High	~10–50 nm	Tracks transient oligomers in live cells
PICUP + SDS‐PAGE/WB	Low to medium	Medium	Medium	Band‐level / qualitative	Detects oligomer size/states; possibility of cross‐linked artifacts
X‐ray crystallography	High (for stable species)	None	Low	Atomic (~2 Å) (Laganowsky et al., 2012; Spencer et al., 2014; Stroud et al., 2012)	Only for very stable oligomers, needs crystal formation and excludes disordered states
SAXS/SEC‐SAXS	Low	Medium	Low to medium	~10–30 Å (Schroer & Svergun, 2018)	Solution‐state size/shape properties (MW, Rg, Dmax; ensemble modeling). Sensitive to polydispersity/heterogeneity; often coupled to SEC; complements NMR/native MS/cryo‐EM
MD simulations	Medium	High	Indirect	Atomistic (in silico)	Requires validation with experimental data
Organoids/3D cell cultures	Low	Medium	High	Cellular‐scale	Biological relevance; functional phenotypes harder to quantify
Mouse models	Low	Low	High	System‐level	Captures behavior/pathology; results may not hold when translated to humans
In‐cell NMR	Medium	High	High	Residue‐specific	Technically demanding; suitable for soluble or stabilized proteins

^a^ High, medium, and low give qualitative assessment of the method for studies of structural polymorphism, transiency, and biological relevance of oligomeric structures.

^b^ These are only rough estimates of the resolution, summarizing the most commonly achievable resolution with this method.

Among high‐resolution biophysical methods, NMR uniquely offers versatile, residue‐specific insights into β‐sheet registry and conformational diversity for assessing polymorphism. Currently, cryo‐EM excels for amyloid fibrils, but remains challenging for oligomers. The small size, structural heterogeneity, and low contrast of oligomers complicate single‐particle averaging. Although methods are improving through better 2D/3D classification and cryo‐ET with subtomogram averaging, the widespread application of cryo‐EM to oligomers remains limited due to heterogeneity of oligomeric samples. X‐ray crystallography has provided a few high‐resolution structures of the oligomers, but faces the challenge of sample crystallization. AFM, advanced fluorescence imaging (e.g., SMLM, TIRF), PICUP, and FRET are effective for studying early oligomeric intermediates, but typically provide low‐resolution data. These methods can detect low‐abundance, short‐lived species both in vitro and in cellular environments. In‐cell NMR and time‐resolved solution NMR methods enable in situ monitoring of conformational exchange and post‐translational modification events. The NMR techniques specifically improved our ability to study oligomer dynamics across relevant timescales.

Cellular models, ranging from primary neurons and engineered cell lines to human‐derived organoids, have provided mechanistic insights into toxic gain‐of‐function. However, the selection of models must be done with caution, as cellular heterogeneity, limited maturity of organoids, and variability in differentiation protocols may produce artifacts and lead to mixed findings. We should also prioritize models that accurately replicate human diseases (patient‐derived iPSC neurons, organoids, and in vivo reporters) and enable real‐time monitoring of oligomer activity (e.g., FRET biosensors, live‐cell super‐resolution imaging). Hybrid methods, such as CLEM combined with immunolabeling or fluorescent tracking in situ, have allowed the correlation of their structural properties with biological relevance.

Most biophysical and biochemical techniques probe only a single property of an oligomer sample. Relying on any one “view” can therefore introduce methodological bias and lead to an incomplete, or even misleading, interpretation of oligomer composition and behavior. For example, spectroscopies report a population mean and cannot resolve minority subpopulations that may dominate toxicity. Crosslinking, chemical trapping, or freezing to stabilize oligomers can enrich for kinetically stable species but may miss transient intermediates. Furthermore, techniques such as AFM and TEM, which require sample adherence to mica or grids, can preferentially reveal “sticky” assemblies with affinity for the immobilization surfaces. Similarly, dyes and antibodies preferentially detect β‐rich or epitope‐accessible states, potentially missing alternative toxic conformers. A common limitation of in vitro experiments is the absence of cellular complexity, and concentrations used are often higher than physiologically relevant levels. This can result in accelerated aggregation and the detection of states that are not common in vivo. At the same time, complex biological matrices (cell lysates, CSF, plasma, brain tissue) can reduce sensitivity, and harsh extraction reagents can alter their properties.

Therefore, a multi‐technique orthogonal approach should be prioritized to assess the unique properties of the oligomers simultaneously and to cross‐reference data from different methods. Such approaches revealed that oligomer populations are structurally polymorphic and showed that oligomeric assemblies can be locally stable, transient, or condition‐dependent. For example, oligomers can form via distinct pathways, such as liquid–liquid phase separation (LLPS) and β‐barrel or pore‐like assemblies. The resulting oligomers were reported to have different structural and phenotypic properties. (Tang et al., 2025). Additionally, a growing body of evidence suggests that early populations, rather than the end‐stage inclusions, may be most bioactive and hence most relevant mechanistically and diagnostically. Consistent with this, a recent tissue‐scale platform combining autofluorescence suppression and single‐molecule fluorescence microscopy enabled direct visualization and quantification of nanoscale α‐synuclein assemblies in human post‐mortem brain tissue, analyzing approximately 1.2 million aggregates and revealing a disease‐specific shift in a small (nanoscale) subpopulation (Andrews et al., 2025). Such multi‐experimental settings reveal the power of direct in situ detection as a way to gain a complete picture of oligomer landscapes and to reconcile controversial evidence obtained by indirect inference from tissue/cellular extracts or model systems.

What is ahead? We still lack routine approaches to obtain residue‐level structural information on transient oligomers in situ at near‐endogenous concentrations, as well as direct, quantitative mapping of oligomer population distributions and their respective cellular phenotypes. Similarly, reconciling in vitro‐defined oligomer “types” with the assemblies present in living systems remains challenging, as extraction and purification can alter oligomers. Hence, important steps toward addressing these challenges include increasing sensitivity while reducing data‐collection time and carrying out experiments simultaneously in biologically relevant model systems. To gain a true view of oligomeric species, developing methods that focus on quantifying oligomer populations and reporting on the distribution of structural polymorphs should be given a high priority. New approaches, such as nanopore‐based sensing, in which individual oligomers produce characteristic ionic‐current signatures as they pass through or interact with a nanoscale pore, should be further developed to enable more accessible, high‐throughput measurements of oligomer size distributions and conformational heterogeneity (Horne et al., 2025). Combining this approach with in situ imaging (Andrews et al., 2025) and linking oligomer formation with pathogenic outcomes (Tang et al., 2025).

Beyond the experimental strategies discussed here, computational approaches, especially machine learning, hold exciting potential for predicting protein–protein interactions, toxic phenotypes, and system‐level behaviors. Utilization of this potential requires large, curated datasets and standardized workflows to enable biological validation in physiologically relevant models. Integrative, AI‐assisted modeling that fuses multiscale measurements (structural, biophysical, and cellular readouts) should also be incorporated to gain a more accurate view of oligomer populations and polymorphs and their contribution to toxicity.

The development of such integrated approaches can shift the focus from “which method is correct?” to “which subset of the oligomer populations is being studied, and what is the connection between the distinct oligomer populations and the specific cellular toxicity?” A deeper understanding of oligomer‐specific toxic pathways is fundamental for designing targeted therapeutics that distinguish between causal species and downstream byproducts.

## AUTHOR CONTRIBUTIONS

**Magdalena I. Ivanova:** Conceptualization; investigation; writing – original draft; writing – review and editing. **Carmelo La Rosa:** Conceptualization; investigation; writing – original draft; writing – review and editing. **Ayyalusamy Ramamoorthy:** Conceptualization; investigation; funding acquisition; writing – original draft; writing – review and editing.

## CONFLICT OF INTEREST STATEMENT

The authors declare no conflicts of interest.

## Data Availability

Data sharing not applicable to this article as no datasets were generated or analysed during the current study.
